# Endovascular Treatment of Thoracic Aortic Aneurysm Causing Life-Threatening Hemoptysis: Two Case Reports

**DOI:** 10.1155/2018/7014170

**Published:** 2018-05-15

**Authors:** Şükrü Oğuz, Süleyman Bekirçavuşoğlu, Zerrin Pulathan

**Affiliations:** ^1^Department of Radiology, Farabi Hospital, Karadeniz Technical University, Trabzon, Turkey; ^2^Department of Cardiovascular Surgery, Farabi Hospital, Karadeniz Technical University, Trabzon, Turkey

## Abstract

**Purpose:**

To describe two patients presenting life-threatening hemoptysis with saccular thoracic aortic aneurysm penetrating lung parenchyma and its endovascular treatment.

**Case Report:**

We present two cases of 73- and 74-year-old men with massive hemoptysis secondary to saccular thoracic aortic aneurysm ruptured lung parenchyma who were successfully treated with endovascular approach with 3rd month's imaging follow-up.

**Conclusion:**

Thoracic aortic aneurysm is one of the rarest causes of hemoptysis and thoracic endovascular aortic repair (TEVAR) and can be used for an effective and problem-solving treatment approach.

## 1. Introduction

Hemoptysis is a common clinical self-limiting symptom; however, it also requires further questioning. It has many causes and the most common causes are acute and chronic bronchiectasis, tuberculosis, fungal infections, and cancer [1]. However, thoracic aortic aneurysm (TAA) is one of the rarest causes, and hemoptysis is a very rare symptom for TAA. It results from penetrating the tracheobronchial tree; however, rupturing lung parenchyma is even a rarer situation. We present two cases with saccular thoracic aortic aneurysm which ruptured lung parenchyma that were presenting with massive hemoptysis and treated with thoracic endovascular aortic repair (TEVAR) along with 3rd month's imaging follow-up.

## 2. Case Reports

**Figure 1:**
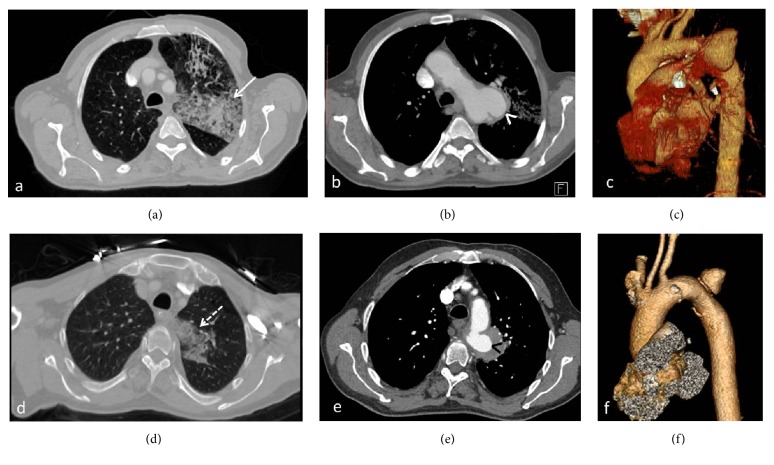
Case 1 (a–c). Case 2 (d–f). Contrast-enhanced CT and volume rendering technique (VRT) images revealed parenchymal hemorrhages in the left upper lungs (white arrow in (a), dashed arrow in (d)) and saccular aneurysms located proximal to the descending thoracic aorta (white arrow head in (b) and black arrow head in (e)).

**Figure 2:**
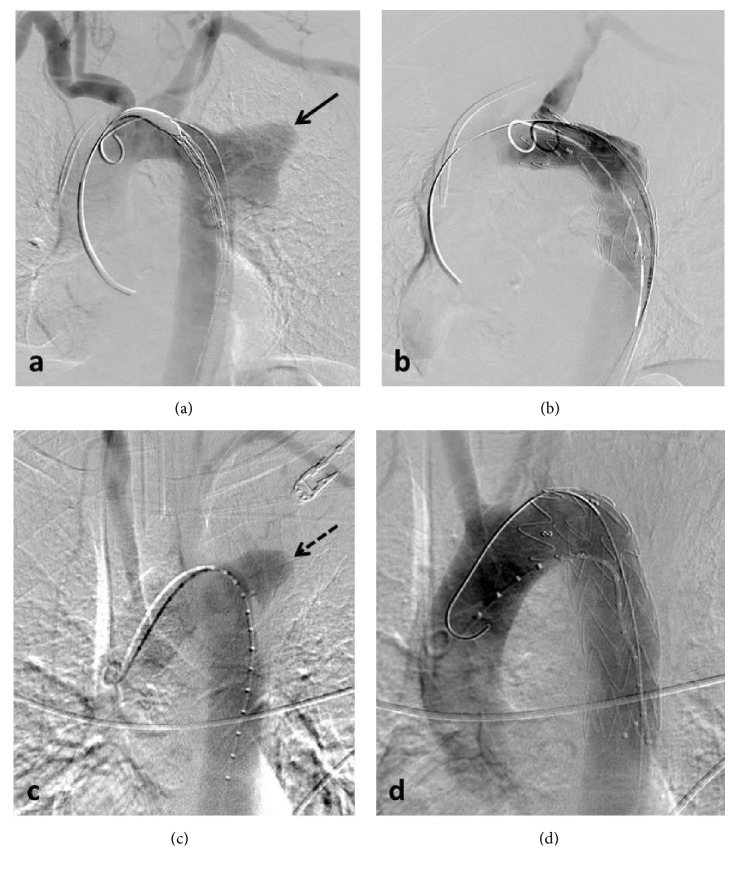
Case 1 (a, b). Case 2 (c, d). Intraoperative digital subtraction angiography images show saccular wide neck aneurysms located proximal to the descending thoracic aorta (black arrow in (a), dashed arrow in (c)) with complete exclusion of the aneurysms after stent-graft deployment (b, d).

**Figure 3:**
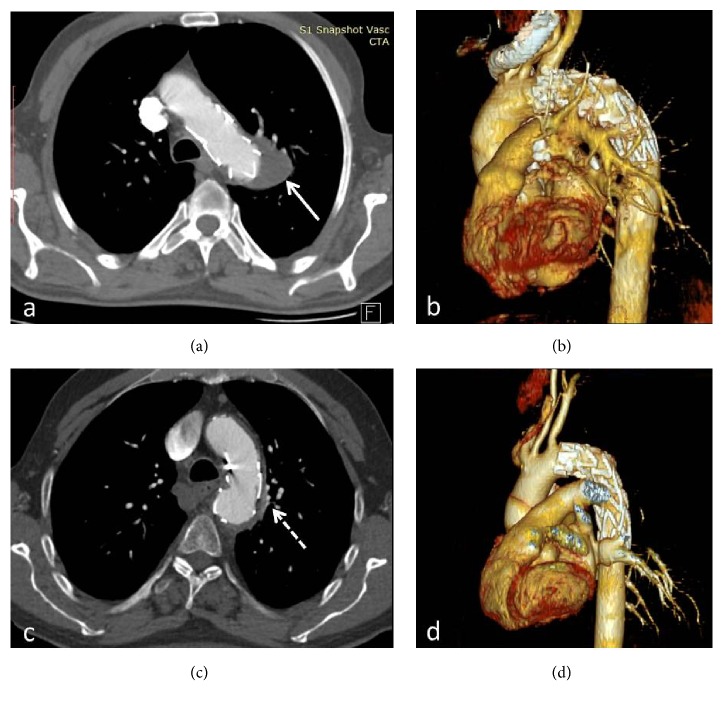
Case 1 (a, b). Case 2 (c, d). Contrast-enhanced CT and VRT images show complete occlusion of the aneurysm with thrombosis and shrinkage, especially in case 2 (white arrow in (a), dashed arrow in (d)).

Two patients—a 73-year-old man (Case 1) and a 74-year-old man (Case 2)—presented with massive hemoptysis. Case 1 was admitted to the emergency service with massive hemorrhage from his mouth and nose. In his past history he had hypertension which had been known for 1 year, 30 pack years of cigarette smoking, and intermittent chest pain for the last 2 years. On examination, coarse crackles were present in the left upper lung zone, his blood pressure was 100/50 mmHg, and heart rate was 72 beats per minute. The hemoglobin level was 7 g/dL and C-reactive protein was 2.39 mg/dL. Case 2 was admitted to the emergency service with massive hemorrhage from his mouth. In his past history, he had hypertension and chronic obstructive lung disease, with 25 pack years of cigarette smoking. On examination, his blood pressure was 130/60 mmHg and heart rate was 138 beats per minute. The hemoglobin level was 9.2 g/dL and C-reactive protein was 10.4 mg/dL. The blood cultures were negative in both patients on admission. Patients were examined with contrast-enhanced thorax computed tomography (CT) for etiologic investigation and treatment planning. For case 1, CT exam revealed a 28 × 40 × 46 mm saccular TAA penetrating ruptured into the left lung upper lobe located behind the origin of the subclavian artery and, for case 2, a 22 × 24 × 26 mm saccular TAA with same futures; however, active contrast extravasation was not observed. Parenchymal hemorrhage was also observed in the left upper lung lobe in two patients especially for case 1 (Figure 1). Both patients underwent emergency TEVAR due to massive hemoptysis secondary to TAA. Under general anesthesia, right femoral artery was explored. Following diagnostic angiograms, Talent™ Stent-Graft System (World Medical, a division of Medtronic Vascular; Sunrise, Fla) was deployed to cover neck of aneurysm by aligning the subclavian artery origin, size 34/34 × 100 mm and 32/32 × 100 mm, respectively. Angiograms showed complete occlusion of the aneurysms and the procedures were terminated (Figure 2). Both patients were given a nonspecific treatment for possible infection prophylaxis during the following week. Contrast-enhanced thoracic CT examinations after 3 months showed that the aneurysms were totally occluded with thrombosis and the size of the aneurysms was significantly reduced and the lung parenchyma hemorrhages also disappeared (Figure 3).

## 3. Discussion

Hemoptysis is a common symptom in clinical practice. It is usually self-limiting and it has many causes which are acute and chronic bronchiectasis, tuberculosis, fungal infections, and cancer [1]. It should be investigated; particularly massive life-threatening hemoptysis requires a thorough clinical and radiological evaluation. Aortobronchial fistula is one of the most dangerous causes of life-threatening massive hemoptysis. Most reported cases of aortobronchial fistula are postoperative, after the implantation of a prosthetic vascular graft in the thoracic aorta. However, the aortobronchial fistula secondary to thoracic aortic aneurysm is an uncommon entity [2].

Thoracic aortic aneurysms develop slowly and usually show a clinically silent course. This makes it difficult to diagnose them. Clinical symptoms change whether rupture or unrupture. Clinical symptoms are pain in the chest, back pain, hoarseness, cough, shortness of breath, or rarely dysphagia in unrupture period; when it was ruptured, patients presented with sharp, sudden pain in the back and chest and difficulty in breathing. If it is ruptured tracheobronchial tree or lung parenchyma, patients will admit likely massive life-threatening hemoptysis. TAA should be kept in mind especially in patients with massive hemoptysis

Descending TAAs account for approximately 30–40% of thoracic aortic aneurysms and fusiform form is common shape of them. Causes of descending TAAs include atherosclerosis, cystic medial necrosis, chronic dissection, and aortitis [3]. Saccular aneurysms are rarely encountered form of the aorta aneurysm and they have a more varied etiology, including aortic infection, degeneration of a penetrating atherosclerotic ulcer, trauma, and previous aortic surgery [4]. We did not find any evidence of infection in our patients. In our two elderly patients with smoking and hypertension history, the etiology of these aneurysms may be secondary to lesions of atherosclerotic ulcerous plaques.

Surgery of aortobronchial fistulas is associated with severe complications and a mortality rate ranging from 25 to 41% [5, 6]. Endovascular stent grafting provides a less invasive approach with reduced morbidity and mortality [7]. In literature a few case series and case reports (some of them are autopsy report) presented about aortobronchial fistula [2, 7–14]. But only a few cases point to direct rupture of the lung parenchyma [12–14]. Fistula develops between aorta and tracheobronchial tree because erosion from the continuous pulsatile pressure may induce necrosis and compression of the tracheobronchial tree by enlargement of the thoracic aortic aneurysm [2].

## 4. Conclusion

In our cases, saccular thoracic aortic aneurysms invaded and were ruptured in the lung parenchyma and they presented with massive hemorrhage. The patients were treated with endovascular approach successfully. Also imaging and clinical follow-ups were clear. TEVAR can be used as an effective and problem-solving treatment approach. Thoracic aortic aneurysm is one of the rarest causes of hemoptysis and hemoptysis for TAA is a very rare symptom. On the other hand, TAA should be kept in mind especially in patients with massive hemoptysis.
